# β-Sitosterol modulates macrophage polarization and attenuates rheumatoid inflammation in mice

**DOI:** 10.1080/13880209.2019.1577461

**Published:** 2019-03-24

**Authors:** Rui Liu, Donglin Hao, Wenya Xu, Jinjin Li, Xiaoru Li, Dong Shen, Kang Sheng, Lin Zhao, Weiwei Xu, Zhongen Gao, Xu Zhao, Qiuhong Liu, Yiting Zhang

**Affiliations:** a Department of Rheumatism, Suzhou Hospital Affiliated to Nanjing University of Chinese Medicine, Suzhou, PR China;; b Department of Rheumatism, Suzhou Science & Technology Town Hospital, Suzhou, PR China

**Keywords:** Rheumatoid arthritis, M2 macrophages, collagen-induced arthritis

## Abstract

**Context:** β-Sitosterol (BS), the primary constituent of plants and vegetables, exhibits multiple biological effects.

**Objective:** This study explores its effect of immune-regulation on macrophages and its potential for rheumatoid arthritis (RA) therapy.

**Materials and methods:***In vitro*, bone marrow-derived macrophages (BMDMs) were treated with 5, 25 and 50 μM BS in the M1 or M2 polarization conditions. *In vivo*, either i.p. injection with 20 or 50 mg/kg BS every 2 d after boost immunization of collagen-induced arthritis (CIA) or adoptive transfer of 2 × 10^6^ BS-treated BMDMs (BS-BMDMs) at the day before CIA were adopted in mice to test the therapeutic effect. IL-10 antibody depletion was used in the period of above treatments to discuss the underlying mechanism.

**Results:** The phenotypes and function of BMDMs showed that 5, 25 and 50 μM BS significantly repressed the M1 polarization and augmented M2 polarization dependent upon concentration. The expression of iNOS, IL-1β, CD86 and MHCII in 25 μM BS-treated M1-polarized BMDMs was reduced by 50.2, 47.1, 87.1 and 31.3%, respectively. In contrast, the expression of arginase-1, IL-10, CD163 and CD206 in 25 μM BS-treated M2-polarized BMDMs was increased by 65.6, 107.4, 23.5 and 51.3%, respectively. In CIA mice, either i.p. injection with BS or adoptive transfer of BS-BMDMs could alleviate the symptoms of ankle swelling (vehicle group: 3.13 ± 0.102 mm; 20 mg/kg BS group: 2.64 ± 0.043 mm; 50 mg/kg BS group: 2.36 ± 0.084 mm; BMDMs group: 3.09 ± 0.174 mm; BS-BMDMs group: 2.43 ± 0.042 mm), reduce the levels of collagen-specific antibodies (IgG and IgG1, but not IgG2c, *p* < 0.05) and inhibit the production of pro-inflammatory cytokines (*p* < 0.05). Depletion of IL-10 counteracted the effect of BS treatment (α-IL-10 *vs.* RatIgG1, *p* < 0.01 on day 16), highlighting the role of IL-10 in the anti-inflammatory response.

**Conclusions:** These results suggested that BS could modulate the functions of macrophages and might be a promising agent for RA therapy.

## Introduction

Rheumatoid arthritis (RA) is a chronic autoimmune disease that affects approximately 1% of the human population. Hyperplasia of synovial tissues, chronic inflammation and progressive articular destruction were generally found in RA patients (Feldmann et al. 1996; Firestein and Mcinnes 2017). The development of RA involves multiple steps, leading to the complex pathogenesis (Mcinnes and Schett 2011). In the pre-RA stage, hypersensitive innate immune system and abnormal T/B-cell signal in mucosal immune system reach a threshold value that initiates a series of adaptive immune responses *via* the recognition of citrulline residues (Scher et al. 2013; Konig et al. 2016). Then a chronic inflammation occurs in the joint and establishes synovitis, which contains abundant activated immune cells, causing progressive cartilage tissue damage.

Innate immune cells, such as monocytes, macrophages and neutrophils, were observed in the joints of RA patients (Van den Berg and Van Lent 1996; Apel et al. 2018). During this disease, substantially activated monocytes increase in blood and migrate along with chemokine gradient to infiltrate into the joint (Mulherin et al. 1996). In the inflammatory joint, these recently infiltrating monocytes differentiate into pro-inflammatory macrophages, which correlate with articular destruction (Yanni et al. 1994). Indeed, macrophages within RA tissue are highly activated to enhance local inflammation, thereby accelerating the development of RA. Several available drugs, such as Etanercept^®^, reduce the number of macrophages in the synovial membrane (Genovese et al. 2002). In comparison, macrophages within tumour facilitate to the establishment of microenvironment of immunosuppression, leading to the exhaustion of cytotoxic lymphocytes (Komohara et al. 2016). Given that macrophages have the bidirectional roles in the inflammatory response, targeting macrophage to repress inflammatory response might be a promising strategy for RA therapy.

β-Sitosterol (BS) is the main constituent of plants and vegetable with various activities (Wang et al. 2012), including anti-hyperlipidemia (Nazeam et al. 2018), anti-inflammation (Lampronti et al. 2017) and anti-tumour (Moon et al. 2008). Several studies also suggested that BS could use as an anti-bacterial agent and possess the ability to protect the gastric mucosa from acetic acid- or aspirin-induced damage (Arrieta et al. 2003). Attractively, BS as a common constituent of human daily diet was easy to ingest from food (Bradford and Awad 2010). In mice model of experimental colitis, mice treated with stigmasterol and BS developed less severity of mucosal colitis (Feng et al. 2017). Additionally, these phytosterols also showed the effect to protect against non-alcoholic fatty liver disease (Feng et al. 2018). However, the effect of BS in RA remained largely unexplored.

In this study, we investigated the function of BS, as well as BS-treated BMDMs (BS-BMDMs). *In vitro*, BS significantly repressed the BMDMs towards to the M1 polarization but augmented the M2 polarization In the CIA C57BL/6 mice model, BS treatment and BS-BMDMs both showed effects to impede the CIA response which was partly dependent on the IL-10 production.

## Materials and methods

### Reagent

BS (synthetic, 95% purity) was purchased from Sigma-Aldrich. BS was first dissolved in absolute ethanol, followed by dissolved in 0.5% cyclodextrin solution. The absolute ethanol was removed by evaporation.

### Bone marrow-derived macrophages culture

Bone marrow-derived macrophages (BMDMs) were prepared as previously described (Lin and Gordon 1979). Briefly, bone marrow cells were isolated from the femur of 6-week-old male C57BL/6 mice. Red blood cells were removed by Red Blood Cell Lysis Buffer (Solarbio, Beijing, China). After washed, the cell suspension was passed a 40 μm cell strainer and plated in a 6-well plate at a density of 2 × 10^6^ with 10 ng/mL macrophage colony-stimulating factor (M-CSF, ProteinTech, Rosemont, IL) for 7 d. The culture medium was replaced every 2 d. BS was added into the medium in the last day of culture to induced BS-BMDMs. For M1 or M2 macrophage polarizations, BMDMs or BS-BMDMs were further stimulated with 10 ng/mL IFN-γ or IL-4 (ProteinTech, Rosemont, IL). A day later, cells were collected for RNA extraction. Alternatively, 2 d later, flow cytometry analysis was performed.

### FACS assays

Cells were harvested and washed with phosphate buffer solution (PBS). Next, cells were first stained with purified anti-mouse CD16/CD32 antibody to block Fc receptor, followed by staining with APC-conjugated anti-mouse CD11b and FITC-conjugated anti-mouse F4/80 antibodies to analyse the purity of BMDMs. For M1 or M2 macrophage phenotype analysis, activated BMDMs were stained with anti-mouse CD16/CD32 and then stained with PE-conjugated anti-mouse CD86 and APC-conjugated anti-mouse MHCII antibodies, or PE-conjugated anti-mouse CD206 and APC-conjugated anti-mouse CD163 antibodies, respectively. Cells were washed three times with FACS buffer and re-suspended in 250 μL FACS buffer. The flow cytometry analysis was performed on a BD Flow Cytometer (BD Biosciences, Franklin Lakes, NJ).

### Analysis of gene expressions

The culture medium was removed and the plate was washed three times with cold PBS. TRIZOL reagent (1 mL) was added into each well to extract total RNA. Quantitative real-time PCR (Q-PCR) was performed with the SYBR qPCR Kit under the guideline of manufacturer’s introduction. The primers used in this study are listed in Table 1.

**Table 1.** Primer pairs.

Gene	Sequences (5' to 3')
*F-GAPDH*	ATGACTCTACCCACGGCAAG
*R-GAPDH*	GGAAGATGGTGATGGGTTTC
*F-Arg-1*	GGAATCTGCATGGGCAACCTGTGT
*R-Arg-1*	AGGGTCTACGTCTCGCAAGCCA
*F-IL-10*	CATTCCATCCGGGGTGACAA
*R-IL-10*	GTAGATGCCGGGTGGTTCAA
*F-NOS2*	GGCAGCCTGTGAGACCTTTG
*R-NOS2*	GCATTGGAAGTGAAGCGTTTC
*F-IL-1β*	CAACCAACAAGTGATATTCTCCATG
*R-IL-1β*	GATCCACACTCTCCAGCTGCA

### Animal experiment

Six-week-old Male C57BL/6 mice were purchased from Nanjing Biomedical Research Institute of Nanjing University. All protocols of the animal experiment were approved by the Committee on Laboratory Animal Care of the Nanjing University of Chinese Medicine. For collagen-induced arthritis (CIA) induction, mice were first subcutaneously immunized with 100 μL emulsion prepared by chicken type II collagen (CII) and complete Freund’s adjuvant (Sigma-Aldrich, St. Louis, MO) in the root of tail. Three weeks later, 100 μL emulsion prepared by CII and incomplete Freund’s adjuvant (Sigma-Aldrich, St. Louis, MO) was injected in the same regions. Next, 30 μg LPS (Sigma-Aldrich, St. Louis, MO) was i.p. injected to accelerate the development of CIA in C57/BL6 mice. Other treatments used in this study are described as timetable in Figure. The swelling of hind paw was measured in a 3-d interval. Antibody depletion of IL-10 (anti-mouse IL-10 antibody, BioXcell, West Lebanon, NH) was started at day 0 and 100 μg anti-mouse IL-10 antibody was injected i.p every 3 d for five times.

### Histology

The ankles were collected in 4% paraformaldehyde, decalcified and then embedded in paraffin. The sections were stained with haematoxylin and eosin.

### Cytokine production in serum

Serum was collected and stored at −80 °C. ELISA assays were performed with the manufacturer’s protocols. Mouse IL-1β, IL-6, IL-12 and IL-10 ELISA kits were purchased from BOSTER Biological Technology Co. Ltd (Wuhan, China).

### Collagen-specific antibodies levels in serum

Serum was first diluted 100-fold by PBS containing 0.1% Tween 20 (PBST) and then added into ELISA plates which were pre-coated with 0.5 μg/well chicken CII. After incubated at 37 °C for 1 h, the plates were washed four times, followed by incubation with pre-diluted horseradish peroxidase-conjugated goat anti-mouse IgG, IgG1 and IgG2c antibodies (dilution 1:1000). After washed six times with PBST, the chromogenic reaction was visualized by tetramethylbenzidine (TMB) and stopped by 10% H_2_SO_4_. The plate was read by a Multiskan GO Microplate Reader (Thermo Scientific, Waltham, MA).

### Statistical analysis

To identify a significant difference, unpaired Student’s *t*-tests or Kruskal − Wallis tests were applied. Data were processed and presented by GraphPad version 5.0 software (GraphPad Software, La Jolla, CA). Error bars showed standard deviations.

## Results

### BS-induced macrophage M2 polarization

**Figure 1.**
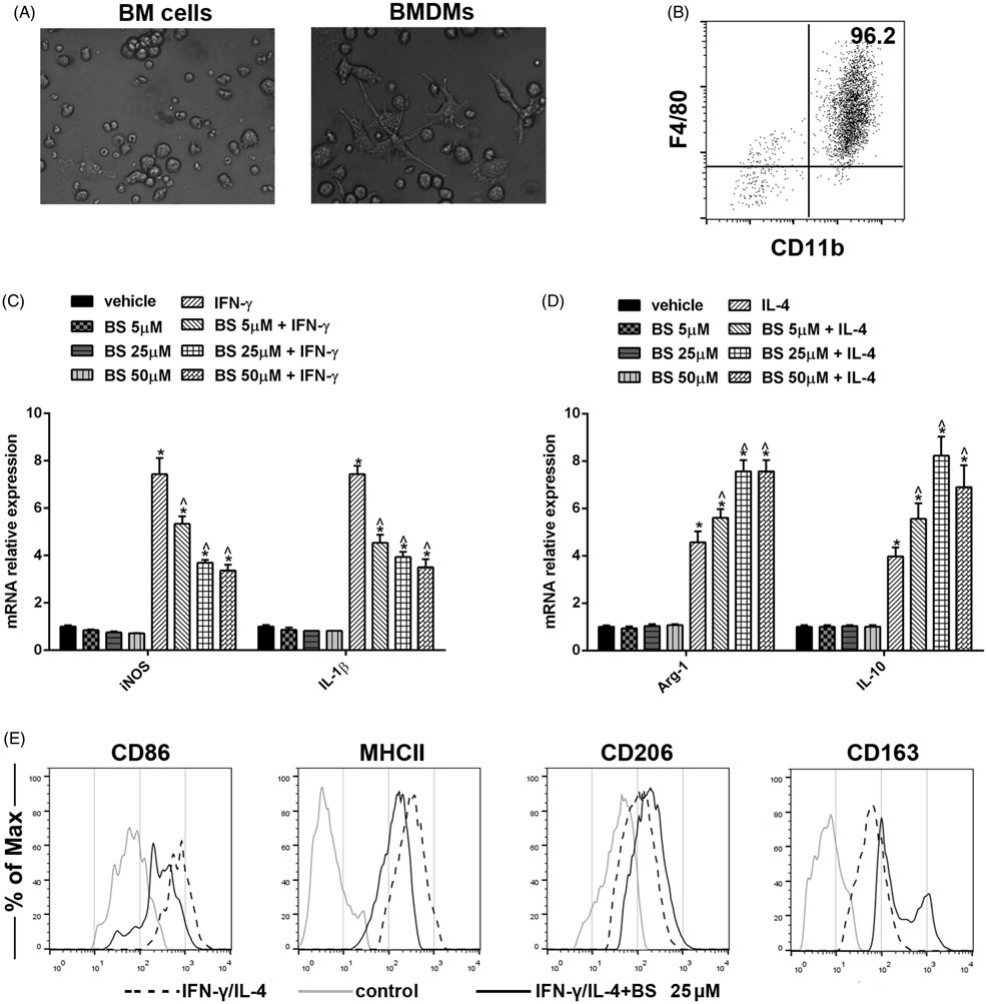
BS altered macrophage phenotypes in the polarization conditions. Bone marrow cells were isolated and cultured with M-CSF for 7 d (A). The adherent cells were collected and the purity was analysed by the macrophage markers CD11b and F4/80 (B). The mRNA levels of arginine-1 and IL-10 in M1 polarization condition (C) or iNOS and IL-1β in M2 polarization condition (D) were analysed by quantitative RT-PCR and the relative expression were normalized to the mRNA level of GAPDH. (E) The expression of M1 macrophage markers CD86 and MHCII or M2 macrophage markers CD206 and CD163 were analysed by flow cytometry. Data were shown as mean and SEM. The experiments were performed at least two times. *p < 0.05.

Macrophages are critical inducers of the inflammatory response. Previously, BS has been reported to impede the inflammatory response in LPS-activated macrophage models. Here, we tried to explore the impact of BS on the macrophage polarization. The bone marrow cells were isolated from the femur and cultured in the presence of M-CSF for 7 d. As shown in Figure 1(A,B), BMDMs demonstrated the adherent morphology after 7 d of culture and expressed CD11b and macrophage marker F4/80. To investigate BS on macrophage polarization, 5, 25 or 50 μM BS was added into the culture at last day of culture. The adherent cells were collected, re-plated and stimulated with 10 ng/mL IFN-γ or IL-4 for another day. Cells were harvested to extract total RNA. The results of quantitative RT-PCR showed that in the presence of IFN-γ, iNOS and IL-1β produced by 25 μM BS-BMDMs reduced 50.2 and 47.1%, while these BMDMs expressed ∼0.5-fold increase of arginase-1 and 1-fold increase of IL-10 upon the stimulation of IL-4, as compared with BMDMs treating with vehicle (Figure 1(C,D)). This might suggest that BS treatment could impede M1 but facilitate M2 macrophage polarization. To affirm this, we measured the M1 (CD86/MHCII) or M2 (CD206/CD163) surface makers in 25 μM BS-BMDMs after stimulation. As excepted, as compared with M1 or M2 macrophages, CD86 and MHCII expressed by BS-BMDMs reduced 87.1 and 31.3% in M1 polarization condition, while upon stimulation of IL-4 they expressed 23.5% increase of CD206 and 51.3% increase of CD163 (Figure 1(E)). Thus, these results indicated that BS could impede the M1 but promote M2 polarization of macrophages.

### BS treatment repressed collagen-induced arthritis, collagen-specific antibodies and pro-inflammatory cytokine production in mice

**Figure 2.**
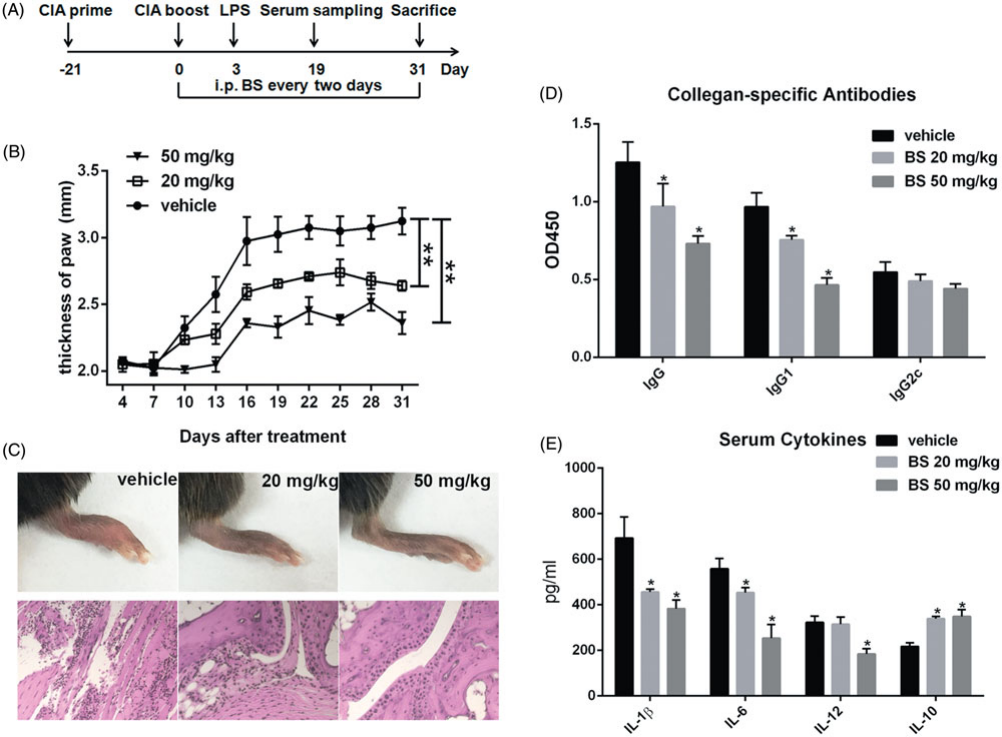
BS treatment repressed collagen-induced arthritis, collagen-specific antibodies and pro-inflammatory cytokine production in mice. (A) Timeline of animal experiment. (B) The swelling of hind paw was measured every three days using a vernier caliper. (C) The H&E staining of hind paws. (D) The collagen-specific IgG, IgG1 and IgG2c antibodies were detected by ELISA at a 1:100 dilution of serum. (E) The levels of serum IL-1β, IL-6, IL-12 and IL-10 were measured by ELISA. Data were collected from two individual experiments, and shown as mean and SEM. *N* = 6–8. *p < 0.05 and **p < 0.01.

To investigate the effect of BS on the RA therapy, the CIA mice were injected i.p. every 2 d with 20 or 50 mg/kg BS. The timeline of the animal experiment is shown in Figure 2(A). The prime and boost immunizations of CIA induction were performed on day −21 and day 0, respectively. BS treatment was started at day 0. 30 μg LPS was injected i.p. at day 3 to enhance CIA response in the C57BL/6 mice. A day later, the thickness of hind paw of these mice was followed. The swelling of ankle was peaked at day 19, and either 20 or 50 mg/kg BS could repress the swelling of hind paw (vehicle group: 3.13 ± 0.102 mm; 20 mg/kg BS group: 2.64 ± 0.043 mm, *p* < 0.01; 50 mg/kg BS group: 2.36 ± 0.084 mm, *p* < 0.01), which was consistent with less inflammatory degree measured by histology (Figure 2(B,C)).

The CIA model is characterized by a potent antigen-specific humoural immune response. Next, we measured the levels of collagen-specific antibodies in serum on day 19. The results showed at a 100-fold dilution that BS treatment significantly inhibited the production of collagen-specific IgG and IgG1 (*p* < 0.05) but not IgG2c, suggesting a dampening humoural immune response (Figure 2(D)). For the cellular immune response, the levels of IL-1β, IL-6, IL-12 and IL-10 were also measured in serum. As compared with the vehicle group, decreased levels of IL-1β, IL-6, IL-12 and increased level of IL-10 were observed in the mice that treated with 50 mg/kg BS (*p* < 0.05, Figure 2(E)). Altogether, these results suggested that BS could repress the pathological alteration of CIA through the inhibition of humoural and cellular immune response.

### Transfer of BS-treated BMDMs repressed CIA response in mice

**Figure 3.**
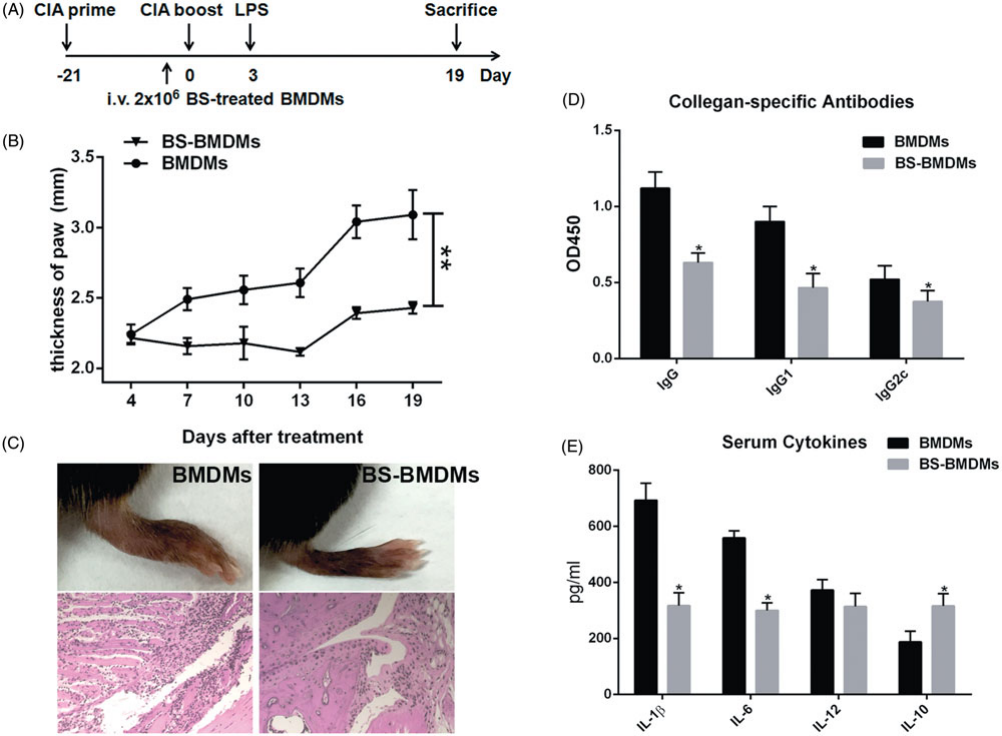
BS-treated BMDMs alleviated collagen-induced arthritis in mice. The CIA was induced as above described. (A) Timeline of animal experiment. 2 × 10^6^ BS-BMDMs or control BMDMs were transferred through tail vein at day −1. (B) The swelling of hind paw was measured every 3 d using a Vernier caliper. (C) The H&E staining of hind paws. (D) The collagen-specific IgG, IgG1 and IgG2c antibodies were detected by ELISA at a 1:100 dilution of serum. (E) The levels of serum IL-1β, IL-6, IL-12 and IL-10 were measured by ELISA. Data were collected from two individual experiments, and shown as mean and SEM. *N* = 5. *p < 0.05 and **p < 0.01.

Some macrophages show strong capabilities of immune-regulation in the tumour microenvironment and chronic inflammatory diseases. As BS-BMDMs facilitated the M2 phenotype, we investigated whether these cells could be used for the RA therapy. Of 2 × 10^6^ BMDMs or BMDMs treating with 25 μM BS were transferred into the mice at day −1. The booster immunization of CIA induction was performed on day 0 and 30 μg LPS was injected at day 3 (Figure 3(A)). The swelling of hind paw was followed, and all these mice were sacrificed at day 19. We found that as compared with the recipients of BMDMs, the recipients of BS-BMDMs showed significantly mitigation of ankle swelling (BMDMs group: 3.09 ± 0.174 mm; BS-BMDMs group: 2.43 ± 0.042 mm, *p* < 0.01) and synovial inflammation (Figure 3(B,C)). In addition, the collagen-specific IgG, IgG1 and IgG2a antibodies, as well as IL-1β and IL-6 were reduced in serum of the recipients of BS-BMDMs (*p* < 0.05, Figure 3(D,E)). Of note, again the IL-10 level was increased in serum (*p* < 0.05). These results indicated that the BS-BMDMs also showed the ability to repress CIA response.

### IL-10 was responsible for the anti-inflammatory response induced by BS treatment and BS-BMDMs transfer

**Figure 4.**
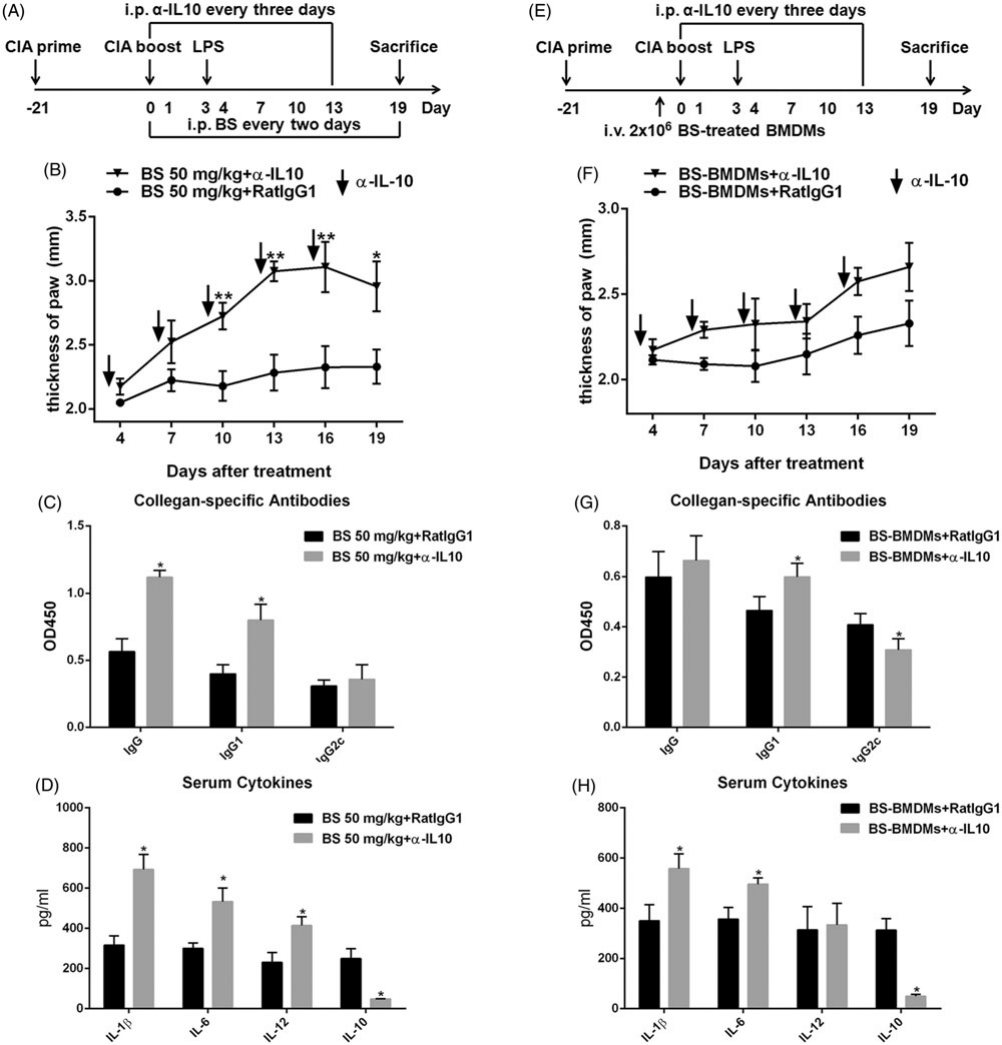
IL-10 was critical for the anti-inflammatory response induced by BS treatment and BS-BMDMs transfer. The CIA induction, BS treatment and BS-BMDMs transfer were performed as above described. 100 μg IL-10 antibody was administered every 3 d and start on day 0. (A, E) Timeline of animal experiment. (B, F) The swelling of hind paw was measured every three days using a vernier caliper. (C, G) The collagen-specific IgG, IgG1 and IgG2c antibodies were detected by ELISA at a 1:100 dilution of serum. (D, H) The levels of serum IL-1β, IL-6, IL-12 and IL-10 were measured by ELISA. Data were shown as mean and SEM. *N* = 5. *p < 0.05 and **p < 0.01.

Next, we investigated how BS treatment or BS-BMDMs repressed the CIA response. Because our results indicated that IL-10, a critical anti-inflammatory cytokine, was increased in both the recipients of BS and BS-BMDMs, we tested whether suppression of CIA response in mice receiving BS or BS-BMDMs was dependent on IL-10 production. To decrease IL-10 *in vivo*, depletion of IL-10 was performed on day 0 to day 15 in a 3-d interval (Figure 4(A)). In mice receiving 50 mg/kg BS, depletion of IL-10 significantly exacerbated the ankle swelling (α-IL-10 group: 3.21 ± 0.31 mm; RatIgG1 group: 2.27 ± 0.12 mm at day 16), in turn discontinuation of anti-IL-10 antibody administration at day 15 to some extent reduced the swelling in hind paw (Figure 4(B)). The collagen-specific antibodies production and serum cytokine levels also suggested that IL-10 was critical for the BS-induced anti-inflammatory response during CIA progression (*p* < 0.05, Figure 4(C,D)). Consistent with this observation, depletion of IL-10 accelerated the progression of CIA in mice that transferred with BS-BMDMs (Figure 4(E,F)). Interestingly, the mice receiving both BS-BMDMs and anti-IL-10 antibody displayed higher IgG1 but lower IgG2c levels, as compared with mice receiving BS-BMDMs and RatIgG1 (*p* < 0.05), even though the IgG levels were approximately equal (Figure 4(G)). This might imply that IL-10 affected the balance of T-helper immune response in these mice. Collectively, these results suggested that BS treatment and BS-BMDMs transfer induced anti-inflammatory responses partly through IL-10.

## Discussion

BS, as a constituent in the daily diet, had versatile activities to impact the normal or aberrant cell activities (Bin Sayeed and Ameen 2015). Previous studies of cancer revealed that BS could induce apoptosis in the lung, leukaemia, stomach and colon cancer cells (Zhao et al. 2009; Baskar et al. 2010; Bradford and Awad 2010), through activation of p53 to evoke caspase cascades and inducing cell cycle arrests (Moon et al. 2008; Rajavel et al. 2018). Recently, the anti-inflammatory effects of BS were highlighted in plant extraction. In LPS-activated J774A.1 macrophages, BS showed anti-inflammatory effect through inactivating STAT1 and NF-κB signal transduction (Valerio and Awad 2011). Importantly, the reduction of pro-inflammatory cytokines but an increase of IL-10 was observed in these macrophages, which was consistent with our observation in BS-BMDMs upon the stimulus with IFN-γ. The similar results were also found in the murine peritoneal macrophages and RAW264.7 macrophages (Choi et al. 2012; Kim et al. 2014). In addition to showing anti-inflammatory effects upon stimulation, we also found that BS could promote the M2 macrophage polarization induced by IL-4, although the underlying mechanism is currently unknown. We speculated that BS might affect the metabolic pathways of activated macrophages to alter their function (Vivancos and Moreno 2008).

The anti-inflammatory effects of BS have been tested in several experimental animal models. For instance, administration of BS could ameliorate the symptoms of dextran sulphate sodium (DSS)-induced colitis in mice receiving a high-fat diet through reduction of colony stimulating factor-1 secretion and inactivation of NF-κB (Kim et al. 2014). In a 2,4-dinitrofluorobenzene-induced atopic dermatitis mice model, BS treatment reduced the local inflammatory response as well as infiltration of inflammatory cells (Han et al. 2014). In this study, we found that BS could significantly reduce the swelling of hind paw (vehicle group: 3.13 ± 0.102 mm; 20 mg/kg BS group: 2.64 ± 0.043 mm; 50 mg/kg BS group: 2.36 ± 0.084 mm), local inflammation, collagen-specific antibodies and serum cytokines in CIA mice. In addition, we transferred the BMDMs that treated with BS and IL-4 into the CIA mice. The progression of CIA was dramatically impeded by the BS-BMDMs transfer.

*In vitro*, BS was reported to induce more IL-10 secretion in macrophages in the stimulation of LPS (Desai et al. 2009; Valerio and Awad 2011). The increased level of anti-inflammatory cytokine IL-10 was also observed in systemic lupus erythematosus mice that were administrated with plant extraction containing BS (Gutierrez Nava et al. 2017). In our models, we found that either BS treatment or BS-BMDMs could induce the elevated IL-10 release in serum (vehicle group: 214 ± 26 pg/mL; BS 20 mg/kg group: 375 ± 14 pg/mL; BS 50 mg/kg group: 383 ± 37 pg/mL; BMDMs group: 198 ± 41 pg/mL; BS-BMDMs group: 337 ± 51 pg/mL), suggesting the pivotal role of IL-10 in BS-induced anti-inflammatory response. Then, we performed anti-IL-10 antibody administration in mice receiving BS or BS-BMDMs transfer. All these mice displayed more potent CIA response along with increased collagen-specific antibodies and serum pro-inflammatory cytokines. In addition, anti-IL-10 antibody administration enhanced the production of collagen-specific IgG1, the IgG subset suggesting the intense inflammatory response. More than IL-10, the immune-regulation effects of BS and BS-BMDMs might be multiple. For example, BS could reduce a series of chemokine expression in the cystic fibrosis bronchial epithelial cells (Lampronti et al. 2017). In the intestine-associated inflammation induced by lipid metabolic disorders, BS treatment inhibited the binding of LPS to toll-like receptor 4 (Kim et al. 2014; Feng et al. 2017), which might be also occurred in our CIA mice model. Moreover, mounting studies about plant extraction demonstrated that BS might be a bioactive constituent to induce the alleviation of the pain in arthritis (De Souza et al. 2012; Bais et al. 2017; Fialho et al. 2017).

RA was a chronic disease that involved the hyperactive release of systemic pro-inflammatory cytokines and aggregated immune cell infiltration into joints of limbs (Smolen et al. 2016). Therefore, therapies that targeted critical cytokines for the expansion of inflammation have been broadly tested in the clinic (Buch et al. 2008). In addition, several antibodies against auto-reactive B or T cells also showed effects to relieve synovium inflammation (De Vita et al. 2002). Using a CIA mice model, we found that BS significantly inhibited the inflammatory cells infiltration. However, the landscape of the CIA response of collagen-specific cellular immune or local acute inflammatory responses was undescribed. In further studies, we will focus on the discrepancy of BS and BS-BMDMs and the underlying mechanisms.

## Conclusions

The results of this study revealed that BS had an effect on the polarization of macrophage to trigger an anti-inflammatory phenotype. Although BS might impact multiple pathways to induce alleviation of CIA, the macrophages and IL-10 were critical in the potential mechanism.
